# Clinical Characteristics and Angiographic Findings of Myocardial Infarction among Marijuana Users and Non-Users

**Published:** 2017-11-22

**Authors:** Justin Lee, Navneet Sharma, Carla Saladini Aponte, Seyed Zaidi, Daniel Fung, Jonathan D Marmur, Moro Salifu, Alyson Myers, William E Lawson, Noelle N Mann, Samy I McFarlane

**Affiliations:** 1,2,3,4,5,6 State University of New York-Downstate Medical Center, Department of Medicine, Divisions of Cardiovascular Medicine and Endocrinology, Brooklyn, NY 11203; 7,8, Stony Brook University Hospital, Department of Medicine, Division of Cardiology, Stony Brook NY 11794

## Abstract

**Background:**

Marijuana use has been increasingly legalized in the United States resulting in substantial rise in the number of users especially in the younger populations. While our group and others had described various metabolic effects of this drug, little is known about its association with acute myocardial infarction (AMI).

**Objective:**

This follow up study presents contemporaneous cohort of non-THC user patients at a single, urban center hospital diagnosed with ST-elevation AMI; highlighting and comparing demographic, clinical, laboratory and angiographic characteristics based on exposure to THC at time of presentation.

**Methods:**

Retrospective chart review of patients with ST-elevation AMI presenting to our inner city hospital Coronary Care Unit over a period of 4 years (December 2013–April 2017).

**Results:**

Of the 10 case subjects studied whom presented with chest pain, EKG evidence of ST-elevation MI (STEMI) with cannabis use, mean age at presentation was 40 years old, which was 10 years younger than our control group with no marijuana use (n = 11, p = 0.107). Of the patients who had marijuana exposure upon admission, 3 (30%) had no known cardiovascular disease (CVD) risk factors (RF) on admission, 1 patient had 3 RF, 4 patients had 2 RF and 2 had 1 CVD risk factor, which included age, diabetes mellitus type 2 (DM2), hypertension, dyslipidemia, smoking status, and family history at time of triage. Patients who were negative for marijuana use had higher number of CVD risk factors present upon admission. ASCVD risk scores were 10% vs. 16% (p = 0.312). In angiographic findings, 100% of the marijuana users had 1 vessel disease compared with 55% in the non-users (p = 0.0351). Severity of stenosis for both groups was averaged at 93% for non-users vs 95% in THC users (p = 0.62414). Collateral vessels were visible during coronary arteriogram in 91% of non-THC users and in only 20% of THC users (p = 0.0019). Furthermore, non-users had 35% higher rate of Rentrop grade 1 collaterals (55% vs. 20%, p = 0.4872). Similar difference was shown in grade 2 collaterals between the two groups with non-users having 36% higher rate (36% vs. 0%, p = 0.0902). Amongst the patients who had collateral circulation present at the time of angiography (Rentrop grade >0), good collaterals (Rentrop grade 2 or 3) were present in 40% of non-THC users, while there was 0% presence of grade 2+ collaterals in THC users (p = 0.5152).

**Conclusion:**

In our study, marijuana use is associated with ST-elevation MI in largely minority population, occurring at a relatively younger age with half of the cases CVD risk free. Additional studies are needed to further characterize this population given the increase in marijuana use.

## Introduction

In the United States, one of the most widely exploited drugs is Marijuana, which is also referred to as cannabis. With swift evolving policies, legalization, and decriminalization, marijuana continues to persist as one of the most debated drugs in the twenty-first century. In 2015, according to the latest survey by National Survey on Drug Use & Health (NSDUH), an estimated 22.2 million Americans aged 12 or older were the consumers of cannabis, accounting for 8.3% of the total population. Although the percentages of the people utilizing marijuana in 2015 were similar to the percentages in 2014, these were significantly higher compared to percentages from 2002 to 2013 [1]. In 2016, a more recent study conducted by Monitoring the Future (MTF) reported a significant increase in daily marijuana use by 7.6% among young adults (aged 19–28); this was the highest level observed in this group since tracking their use initiated 30 years ago, which is three times the level in 1992 [2]. As of January 1, 2017, the use of both recreational and medical cannabis has been legalized in 8 states (Alaska, California, Colorado, Maine, Massachusetts, Nevada, Oregon and Washington) and the District of Columbia in the United States [3]. With the increasing legalization of the drug, the use of marijuana has also been on the rise. Unlike many other drugs, Cannabis has unique chemical complexities, which can further challenge its scientific study. Diverse strains of cannabis plants can produce variable assortment of centrally active substances. The various ratios of these centrally active chemicals then yield numerous central effects, thus leading to countless unpredictable clinical realities [4]. A recent study has suggested that longer-term moderate/heavy marijuana use during early and late 20s is associated with negative health outcomes at age 50 [5]. Recreational use of marijuana is also associated with ethnic identity amongst minority youth population, adding complexity to the extent of drug usage in communities with diverse backgrounds [6]. With the rising use and demand for marijuana, commercial preparations containing synthetic cannabinoids have also rapidly emerged in the recent past. A recent study found that although it is often assumed to be a safe and legal alternative to marijuana, due to its enhanced metabolic toxicity, it should not be considered a safe alternative [7]. In fact, a survey of 80,000 drugs users who utilized synthetic cannabinoids as oppose to tradition cannabis stated a thirty times higher odds of reporting to the emergency departments with symptoms such as nausea, vomiting, breathlessness, hypertension, tachycardia, chest pain, and occasionally acute renal failure [8]. Based on the recent trend towards legalization of marijuana, it is imperative to understand its impact on health outcomes. The scientific evidence regarding the effect of marijuana use on health outcomes is quite limited and is an area of active research.

Cardiovascular disease (CVD) is the number one cause of death in the United States with 1 in every 4 death is attributed to CVD [9]. It includes coronary artery disease (CAD), including myocardial infarction (MI), hypertension, congestive heart failure (CHF), arrhythmias, peripheral vascular disease (PVD) and strokes. Majority (70%) of CVD risk are attributable to modifiable risk factors, such as obesity and cigarette smoking [10]. The effect of marijuana use on CVD is largely unknown.

It should be noted that racial and ethnic differences have been observed in CVD. The prevalence of CVD and its many risk factors is disproportionately higher in black population compared to other ethnic groups [11]. There are many proposed hypotheses as to why these discrepancies may exist, including poor access to health care services, non-adherence to treatment recommendations, inadequate training and environmental and genetic variations [11, 12]. It is also interesting to note that marijuana use was significantly higher amongst black Caribbean adolescents, compared to other ethnicities in a study conducted in an urban setting in UK [13].

In this article, we report a series of acute ST-elevation myocardial infarction (STEMI) events, with comparison of clinical characteristics and angiographic findings between marijuana users and non-users in an urban population with predominantly Black Caribbean representation.

## Methods & Materials

This study is a follow up project from our most recent study of STEMI in marijuana users. For this retrospective study, patients were identified with an SQL server system that looked into the electronic medical record (EMR) at our hospital for this. The server system provided an ad hoc report with the medical record number of 94 adult patients admitted to the cardiac care unit (CCU) with a primary diagnosis of acute ST elevation myocardial infarction (STEMI) from January 2014 to April 2017. We focus here in the characteristics and findings of patients with STEMI negative for marijuana and how do they compare to our previous subjects that had STEMI with urine toxicology positive for cannabinoids.

Our study included: 1. Patients admitted to CCU with a primary diagnosis of acute STEMI, 2. Patients with significant ST segment changes described as 1mm elevation from the J point in at least two contiguous leads. And finally: 3. Only subjects with negative urine toxicology for cannabis at the time of admission were used as control subjects for this study. We excluded patients with the following criteria: 1. Patients with no urine toxicology done during the admission, 2. Patients without coronary angiography. 3. Patients with other reasons for ST elevations besides STEMI (i.e., acute pericarditis).

Once the sample group was determined, baseline characteristics for each subject were collected from the electronic medical record for age, sex, and race. We also obtained vital signs, laboratory values and medical co-morbidities at the time of presentation. Family history for CAD was present if there was documentation in chart of patient’s knowledge of first-degree family members with coronary disease before the age of 65 years old. Medical management provided during hospitalization prior to cardiac catheterization was recorded. All patients underwent cardiac catheterization for diagnostic and or therapeutic reasons. Coronary angiograms were used to study epicardial vessels and determine severity of coronary heart disease. Severe heart disease was present if 70% or more stenosis was present in at least 1 of the 3 main heart vessels. In patients with 50% or more stenosis at the left main artery prior to its bifurcation, this vessel was counted as 2 vessels. Finally, we used the coronary angiograms to determine the presence and degree of collateral coronary circulation to the stenotic vessel. Degree of collaterals was evaluated by the Rentrop criteria. Just like in other studies, grade 0, meant no collaterals; Grade 1, recipient side branch filling only; Grade 2, partial filling of main recipient vessel; and Grade 3, complete filling of main epicardial recipient vessel. Patients with grade 2 or 3 collateral degree were included in good collateral group and patients with grade 0 or 1 collateral degree were included in poor collateral group. All patients had cardiac echocardiography to determine cardiac function.

## Statistical Analysis

Stat plus software was the main software used for data analysis. The calculated averages from this study, non-THC users (control group) and THC users (experimental group) were compared using two tailed statistical t test, assuming no mean difference within them. Null hypothesis, meaning that no difference would be expected between both groups, was rejected if p<0.05, meaning significant difference between the groups was not due by chance alone.

## Results

### Baseline characteristics for THC positive patients

Of the 10 case subjects studied whom presented with chest pain, EKG evidence of ST-elevation MI (STEMI) with cannabis use, mean age at presentation was 40.1 years ± 9.7 SD, ranging from 26 to 59 years old. In total there were 9 male cases and one female case, of them, 8 were Black, 2 Hispanic and 1 White. Of the 10 cases, 3 (30%) had no known cardiovascular disease (CVD) risk factors (RF) on admission, 1 patient had 3 RF, 4 patients had 2 RF and 2 had 1 CVD risk factor, which included age, diabetes mellitus type 2 (DM2), hypertension, dyslipidemia, smoking status, and family history at time of triage. Troponin l (cTnl) peak mean level was 93.5 ± 34.35 ng/ml, range 7.86 – 358.0 ng/ml.

### Baseline Characteristics of THC negative patients

Ninety-seven admissions with a primary diagnosis of STEMI were evaluated as possible study subjects for this project. Table 1 shows baseline characteristics of our control group for the study. Only 11 cases met inclusion criteria with negative urine toxicology for cannabinoids. Majority of patients were male 82%. Our study included 1 white, 8 black, 2 subjects categorize as race other than white or black. The average age of subjects was 49.6 years ± 15.6 SD, ranging from 28 to 70 years old. In 6 out of the 11 cases, urine toxicology was negative for all recreational substances. Urine toxicology was evaluated for other recreational drugs of abuse. Urine toxicology was positive for other substances besides cannabis in 45% of subjects, 4 of them positive for opiates and 3 for benzodiazepines. Of the drugs tested, opiates were the most common drug subjects tested positive (36%), followed by benzodiazepines (27%).

### Risk for Coronary Heart Disease (CAD)

Risk factors for CAD in both groups are shown in table 1. Subjects in both groups had risk factors for ischemic heart disease but the non-THC group had a higher percentage of individuals with HTN (45 %vs 30%), DM (45% vs 10%) and kidney disease (eGFR < 60 ml/(min 1.73 m2)) (18% vs 0%). Serum cholesterol was higher in the THC group than in the non-THC, 210 vs 153 (p= 0.00596). Positive family history was not recorded as being present for any THC group individuals, although it was present in 27% of the non-THC users. A recent smoking history was obtained, 80% in THC users vs only 36% in non-THC users admitted to current smoking status. The most shared comorbidity among non-THC subjects was obesity (present in 73%). The comorbidity most shared among THC users was current tobacco smoking with 80% admitting their use on hospitalization. BMI was higher in non-THC users 31.7 vs 29.4 in non-users, p=0.20189. Risk score assessments for ischemic heart disease both groups were calculated and are available in table 2. Age requirement and lipid panel results to calculate 10- year risk score known as Framingham “Hard” Coronary Heart Disease were met by 7 out of the 11 subjects in the non- THC and 8 out of the 10 THC users. For the non-THC, calculated scores range from 0.0% to 18.8% vs THC users risk score range 1.70% to 21.30%. Mean Framingham risk score for non-users was 6.53% ± 6.26% SD vs THC users average of 9.45% ± 8% SD, p=0.44503. We also calculated the Atherosclerotic cardiovascular disease (ASCVD) 10-year risk score in patients that met age criteria. Five non-THC users met criteria risk calculation criteria and 6 THC users met criteria. In the THC user group 8 of the STEMIs were associated Left Anterior Descending as the site of the culprit lesion, 1 in the Right Coronary Artery, and 1 in the 1st Obtuse Marginal Artery. In the non-THC user group there were 7 lesions in the Left Anterior Descending Artery, 1 in the Left Main Artery, 7 in the Right Coronary Artery, 1 in the 1st Diagonal Artery, 1 in Left Circumflex Artery, and 1 in a Saphenous Venous Graft to the Obtuse Marginal Artery. ASCVD range in non-users ranged from 8.4% to 21.0% with a mean average of 16.32% ± 5.29% SD. THC users ASCVD ranged from 2.70% to 29.20% with a mean average of 10.78% ± 11% SD, p-value between both group equal 0.31248.

### Diagnostic testing and management received during hospitalization prior to undergoing cardiac catheterization

All patients received EKG testing after targeted history was obtained on arrival to the ED. Diagnosis of STEMI was made after ST elevations were recorded on EKG trace with a chief complaint of typical cardiac chest pain. Table 4 shows ST elevations comparison between THC users and non-users. ST elevations for all non-THC users ranged from 1.5 to 4.5 mm with a mean of 2.6 mm ± 0.9 SD. THC users had ST elevations ranging from 2mm to 6mm with a mean of 3mm ± 1.5 SD. Blood samples were collected on all study subjects on arrival to our Emergency Room. Samples were analyzed for basic serum electrolytes and complete blood panel levels. All patients underwent serial troponin I testing on hospital arrival as part of their diagnostic workup. Mean peak troponin I in non-THC was 85.56 ± 73.38 SD ng/ml vs THC users mean of 93.5 ± 108.6 ng/ml, p=0.84819. Table 3 shows the treatment received in both groups prior to cardiac catheterization. All patients in the study, regardless of THC use received aspirin and P2Y12 inhibitors after the diagnosis of STEMI was made. All THC users received heparin infusion prior to cardiac catheterization vs only 64% of non-users received heparin infusion due to comorbidities that prevented infusion. 82% of non-users vs 20% of THC users were already on statin treatment prior to cardiac cath. In non-users, 73% received beta-blockers vs 20% in the THC group.

### Cardiac catheterization findings

Once STEMI was diagnosed, each patient underwent diagnostic and/or therapeutic cardiac catheterization. When the angiograms were evaluated on number of vessels diseased, 100% of the THC user group had 1 vessel disease compared with 55% in the non-users, p=0.0351. Of the Non-THC users, 18% had 2-vessel and 27% had 3-vessel disease. Severity of stenosis for both groups was averaged at 93% for non-users vs 95% in THC users, p= 0.62414. Collateral vessels were visible during coronary arteriogram in 91% of non-THC users and in only 20% of THC users, p=0.0019. We used the Rentrop criteria to assess the degree of collateral circulation, where the degree of recipient artery contrast filling is used to classify collateral vessels: Grade 0, no collaterals; Grade 1, recipient side branch filling only; Grade 2, partial filling of main recipient vessel; and Grade 3, complete filling of main epicardial recipient vessel. Using these measures, we found Rentrop grade 1 collaterals in 20% of marijuana user group, while the rest of the group had zero collaterals. Amongst non-users, the degree of collateral circulation using the Rentrop grade 0, 1, 2 and 3 were 9%, 55%, 36%, and 0% respectively. Non-users had 35% higher rate of grade 1 collaterals (p=0.4872). Similar difference was shown in grade 2 collaterals between the two groups with non-users having 36% higher rate (p=0.0902). Neither group had grade 3 collateral, (p=1.0). Amongst the patients who had collateral circulation present at the time of angiography (Rentrop grade >0), good collaterals (Rentrop grade 2 or 3) were present in 40% of non-THC users, while there was 0% presence of grade 2+ collaterals in THC users (p=0.5152).

In the THC user group 8 of the STEMIs were associated Left Anterior Descending as the site of the culprit lesion, 1 in the Right Coronary Artery, and 1 in the 1st Obtuse Marginal Artery. In the non-THC user group there were 7 lesions in the Left Anterior Descending Artery, 1 in the Left Main Artery, 7 in the Right Coronary Artery, 1 in the 1st Diagonal Artery, 1 in Left Circumflex Artery, and 1 in a Saphenous Venous Graft to the Obtuse Marginal Artery.

Left ventricular ejection fraction (EF) as determined by cardiac echocardiogram in both groups averaged at 43%, p=0.98975.

## Discussion

There was non-significant difference in the age at which MI occurred between THC users and non-users (40.1 ± 9.7 SD vs. 49.6 ± 15.6 SD years). Although statistically non-significant due to small sample size in the study, it is noticeable the gap in the age of onset that suggests there is a potential impact of marijuana smoking on earlier onset of STEMI and other cardiovascular diseases. Higher incidence of MI in younger population should not be left unnoticed because it is considered a rare occurrence in this population despite the higher incidence rate in the African American population (2.2 per 1,000 person years), which was the majority in our study sample (9 of 10 amongst users, 8 of 11 amongst non-users) [14]. This could potentially highlight the early onset of cardiovascular harm of marijuana use.

At cellular level, myocardial damage typically results from the alteration of coronary blood flow, which promotes ischemia and potentially lead to infarction [15].

Recent analysis have shown 3 fold increase in hypertensive death with marijuana use, including cerebrovascular and cardiovascular death. To our knowledge, this is the first study that has attempted to define coronary characteristics in THC users versus non users.

It has been shown that short-term activation of CB_1_ receptor from marijuana use increases the risk of acute MI by 5-folds in the first hour after smoking and then declined rapidly after the initial hour [16]. One possible mechanism for this observed effect is the increase in the concentration of carboxyhemoglobin, which restricts oxygen carrying capacity of red blood cells and thus leads to decreased oxygen supply to myocardium [16]. It is also probable that concomitant increase in heart rate and blood pressure from marijuana use contributes to the mismatch in supply and demand of oxygen to the heart even further by reducing diastolic coronary filling and elevating diastolic coronary pressures, respectively. Therefore, reduced oxygen carrying capacity and potential systemic and coronary vasoconstriction, caused by marijuana use, lead to the reduction in coronary blood flow and increase in myocardial oxygen supply-demand mismatch, resulting in ischemia and infarction [16].

Another possible mechanism in which marijuana usecanleadtoCADisviatheactivationofpro-inflammatory molecules and their pathway in the coronary vasculature. Cannabinoids have shown to potentiate the production of arachidonic acid by causing inflammation that leads to the production of its precursor, 2-Arachidonoylglycerol (2-AG) [17, 18]. Also, THC was found to increase cyclooxygenase 1 and 2 (COX-1, COX-2) levels and causes thromboxane A2 and subsequent prostaglandin production in animal models [19]. These findings suggest that higher levels of pro-inflammatory molecules, such as arachidonic acid and COX in marijuana users may serve as significant factors in the risk of cardiovascular event.

The effect of THC on platelets are not limited to its interaction with arachidonic acid, as it also induces platelet aggregation by potentiating Glycoprotein IIa/IIIb and P-selectin expression via the activation of CB_1_ and CB_2_ receptors [17, 20]. The aforementioned arachidonic acid precursor, 2-AG also potentiates platelet aggregation by inducing phosphorylation of platelet actin molecules through the action of myosin light chain kinase, which subsequently results in platelet conformational change and aggregation. Additionally, the conformation changes result in ATP secretion and 2-AG mediated platelet aggregation [21]. Several clinical trials have also shown the effect of Glycoprotein IIa/IIIb inhibitors on retardation of THC-induced platelet aggregation, further strengthening the argument involving THC mediated platelet aggregation and consequent myocardial ischemia and infarction [22].

The major finding of our investigation was the markedly different angiographic appearance of the myocardium between marijuana users and non-users. Our data suggest that patients experiencing THC associated myocardial infarction have lesser angiographic evidence of atherosclerotic CAD. Amongst the patients who had STEMIs with concurrent marijuana use, 100% of the cohort were found to have 1-vessel disease, whereas in the group of patients with STEMIs that was negative for marijuana use, the prevalence of 1-vessel disease was much lower at 55%. In addition, chronic atherosclerotic sequelae were much more evident in non-marijuana users with 18% of them having 2-vessel disease and 27% with 3-vessel disease. There have been angiographic reports that in THC associated MIs, coronary vasospasm and platelet thrombus formation take place without underlying atherosclerosis [23]. Furthermore, the acuity in THC associated MI was exacerbated by lack of collateral circulation. Our data show significant difference in collateral circulation between marijuana users and non-users, with 80% of marijuana users having Rentrop grade 0 and 20% with Rentrop grade 1, while the non-users had much more extensive development of collateral circulation with only 9% having Rentrop grade 0 and 55% and 36% having grade 1 and 2 collateral circulation, respectively. The lack of collateral circulation observed in THC associated MI could stem from the fact that the individuals did not have underlying chronic CAD as is evident with relatively low atherosclerotic cardiovascular disease (ASCVD) 10-year risk score compared to non-user control group (10.78% ±11.0 SEM vs. 16.32% ± 5.29 SEM, p=0.312). The additives effect of acute thrombus formation with poor collateral circulation could explain the higher peak troponin levels compared to control (93.5 ng/ml ± 34.35 SEM vs. 85.6 ± 73.38, p=0.848) despite minimal underlying CAD. In comparison, majority of the patients with STEMI who had concomitant marijuana use had less comorbidities and less risk factors for cardiovascular disease than their non-user counterpart at the time of diagnosis. There was less incidence of obesity, DM, HLD and family history of CAD. Only significant risk factor for ischemic events that was shared amongst both groups was tobacco smoking, with 80% use in THC group and 40% us in the non-user group. It is difficult to assess the effect of THC in a larger population in a clinical setting, since most our test population had already admitted to marijuana use on admission prior to obtaining the urine sample.

It is interesting to note that marijuana users had relatively lower rates of type II diabetes mellitus (DM2), compared to non-users (10% vs. 45%). This finding is supported by the previous investigation by Muniyappa et al. in the metabolic effect of THC, specifically in glucose sensitivity and insulin secretion by pancreatic β-cells, which suggested that in long-term heavy users of marijuana, THC does not appear to affect glucose sensitivity in peripheral tissues and pancreatic β-cell function, leading to normal glucose tolerance [24].

Moreover, it is interesting to note that obesity prevalence in the study population was 10%. This observation is supported by the results from CARDIA and NHANES III studies suggesting the association between marijuana use and lower BMI with lower abdominal fat content [25, 26]. Recent analysis have shown 3 fold increase in hypertensive death with marijuana use, including cerebrovascular and cardiovascular death. To our knowledge, this is the first study that has attempted to define coronary characteristics in THC users versus non users. However, upon further investigation of relative amount of abdominal fat distribution, a study conducted by Muniyappa et al. has found that marijuana smokers had significantly higher visceral fat content, as opposed to subcutaneous fat, when compared with non-users [24]. This finding is significant because the ratio between visceral adipose tissue and subcutaneous adipose tissue is an independent predictor of cardiovascular events, irrespective of presence of risk factors [27].

Some limitations of our study include the small sample size, confounding factor of tobacco smoking, and predominantly unknown family health history in the study population. Furthermore, the last known time of marijuana use could not be ascertained as this was a retrospective analysis, though all subjects under “marijuana user” group had positive urinary toxicology for THC and negative for other stimulants or substances. Additionally, the chronicity of marijuana use could not be established given the retrospective analysis.

Multiple case reports of ACS after marijuana use have been published in the literature. But evaluation of cardiovascular effects of marijuana is complicated due to concurrent use of other drugs like cocaine, poor quantification, and the presence of multiple chemical compounds in marijuana other than THC. Also, different methods of consumption of marijuana may alter the amount and types of chemicals ingested. For example, blunts are marijuana rolled in tobacco leaves, and joints are rolled in cigarette paper. Therefore, using blunts will produce effects of nicotine in addition to the effects of marijuana. Using joints, on the other hand, results in inhalation of chemicals from the combustion of paper.

In conclusion, despite a number of limiting factors in our study, there seems to be an interesting relationship between marijuana use and its cardiovascular consequence, notably in acute myocardial infarction. The characteristics of acute MI amongst marijuana users in this study show that they occur in younger individuals, with lower pre-existing coronary risk factors when compared with non-users. Most of the individuals suffered from acute thrombus formation in the coronary system. Myocardial damage was likely exacerbated by the lack of collateral circulation, represented by reduced EF and high peak troponin levels. Although it is difficult to claim any causal relationship between marijuana use and acute MI, it is nevertheless very intriguing to find its use associated with MI’s in patients with less number of cardiovascular risk factors. It is important be aware of the potential harms of marijuana use and its effect on the cardiovascular system, both at molecular and clinical levels, given the increased exposure and use of marijuana in the US. Further studies are needed to better understand the interaction between THC, metabolic derangements, inflammation, vasoreactivity and platelet aggregation that result in myocardial ischemia and infarction. Efforts to meticulously characterize patients at risk to suffer from myocardial infarction associated with THC are needed. Additionally, understanding the biochemical pathways involved can help guide therapies. The role of antiplatelets, statins, beta-blockers, calcium channel blockers, angiotensin converting enzyme inhibitor in primary and secondary prevention remains to be seen. Such measure will allow better understanding of the pathophysiology and potential prevention strategies of THC associated myocardial infarction.

## Figures and Tables

**Table 1:** Baseline Characteristics of the Study Population

Baseline characteristics	STEMI (n=11) (%)	STEMI + THC (n=10) (%)	p- value
Age in years at presentation	50 (28–70)	40 (26–59)	0.10737
**Sex**			
Female	18	10	
Male	82	90	
Race			
White	9	10	
Black	73	70	
Hispanic	9	20	
**Blood pressure at presentation**			
Systolic blood pressure	122 ± 22	127 ± 22	0.61189
Diastolic blood pressure	82 ± 16	83 ± 14	0.88282
**Cardiovascular risk factors**			
Dyslipidemia	36	20	
Current tobacco smoking	36	80	
Arterial hypertension	45	30	
Diabetes mellitus	45	10	
Obesity	73	40	
BMI	31.7 ± 4.16	29.4 ± 3.8	0.20189
Total Cholesterol	153 ± 43.3	210 ± 33.3	0.00596
Positive family history of CAD	27	0	
eGFR < 60 ml/(min 1.73 m2)	18	0	
Previous history of MI	36	20	

Values are expressed as (mean ± SD) or as (percentage %)

**Table 2:** Risk for Coronary Heart Disease in the Study Population

Risk for Coronary Heart Disease (CHD)	STEMI	STEMI + THC	p- value
Atherosclerotic Cardiovascular Disease (ASCVD) 10- year risk	16.32% ± 5.29	10.78% ± 11.0	0.31248
Framingham 10- year risk	6.53% ± 6.26	9.45% ± 8.0	0.44503

Values are expressed as (% ± SD)

**Table 3:** Management Received during Hospitalization Prior to Undergoing Cardiac Catheterization

Medical management prior to cardiac catheterization	STEMI (n=11) (%)	STEMI + THC (n=10) (%)
Statin	82	20
Beta Blocker	73	30
ACE inhibitor	55	20
Aldosterone Antagonist	0	10
Calcium Channel Blockers	9	10
Aspirin	100	100
P2Y12 Inhibitors	100	100
Heparin	64	100

Values are expressed as (percentage %)

**Table 4:** Diagnostic Testing Results with Angiographic Findings

Diagnostic findings and angiographic characteristics	STEMI (n=11) (%)	STEMI + THC (n=10) (%)	p- value
Troponin I (peak)	85.6 ± 73.38	93.5 ± 108.6	0.84819
ST Elevation (mm)	2.61	2.9	0.48799
LVEF ( % ± SD)	43 % ± 14	43 % ± 15	0.98975
**Number of significant CAD**			
1-vessel disease	55	100	
2-vessel disease	18	0	
3-vessel disease	27	0	
Lesions identified with more than 70 % stenosis	n=18	n=11	
Stenosis severity (% ± SD)	93 % ± 9*	95 % ± 9*	0.62414
**Collateral circulation assessment**			
Rentrop grade 0	9	80	
Rentrop grade 1	55	20	
Rentrop grade 2	36	0	
Rentrop grade 3	0	0	

Values are expressed as (mean ± SD) or as (percentage %) unless stated otherwise.
